# Supplementary material for: Sex-specific regulatory architecture of pancreatic islets from subjects with and without type 2 diabetes
Source: EMBO J. 2024 Nov 20;43(24):6364–82. doi: 10.1038/s44318-024-00313-z (PMC11649919; doi:10.1038/s44318-024-00313-z)
Supplement: Supplementary file 1 — Appendix [file 44318_2024_313_MOESM1_ESM.pdf]

**Sex-specific regulatory architecture of pancreatic islets from subjects with and without type 2 diabetes**

Mirza Muhammad Fahd Qadir[1,2,3], Ruth M. Elgamal[4,5], Kejing Song[6], Parul Kudtarkar[5], Siva S.V.P Sakamuri[7], Prasad V. Katakam[7], Samir El-Dahr[8], Jay K. Kolls[6], Kyle J. Gaulton[5], Franck Mauvais-Jarvis[1,2,3,9,]

[1]Section of Endocrinology and Metabolism, John W. Deming Department of Medicine, Tulane University School of Medicine, New Orleans, LA, USA

[2]Southeast Louisiana Veterans Health Care System, New Orleans, LA, USA
[3]Tulane Center of Excellence in Sex-Based Biology & Medicine, New Orleans, LA, USA
[4]Biomedical Sciences Graduate Program, University of California, San Diego, La Jolla, CA, USA [5]Department of Pediatrics, University of California, San Diego, La Jolla, CA, USA
[6]Center for Translational Research in Infection and Inflammation, John W. Deming Department of Medicine, Tulane University School of Medicine, New Orleans, LA, USA

[7]Department of Pharmacology, Tulane University School of Medicine, New Orleans, LA, USA
[8]Department of Pediatrics, Tulane University, School of Medicine, New Orleans, LA, USA
[9]Lead contact: fmauvais@tulane.edu

**Appendix Figure S1:** Donor metadata, scRNAseq quality control metrics and network analysis of all non-diabetic cell cells. Page 2.
**Appendix Figure S2:** Differentially expressed genes and pathways in human islet cells across sex and race. Page 4
**Appendix Figure S3:** snATACseq quality control metrics and network analysis of all non-diabetic cells. Page 6
**Appendix Figure S4:** Dynamic insulin and glucagon secretion coupled with islet bioenergetics. Page 8
**Appendix Figure S5:** Dynamic hormone secretion profiles of HPAP type 2 diabetic donors. Page 9 **Appendix Figure S6:** Gene ontology networks in human islet cells from type 2 diabetes donors. Page 10 **Appendix Figure S7:** Cell type-specific, GWAS using DIAMANTE T2D dataset. Page 12

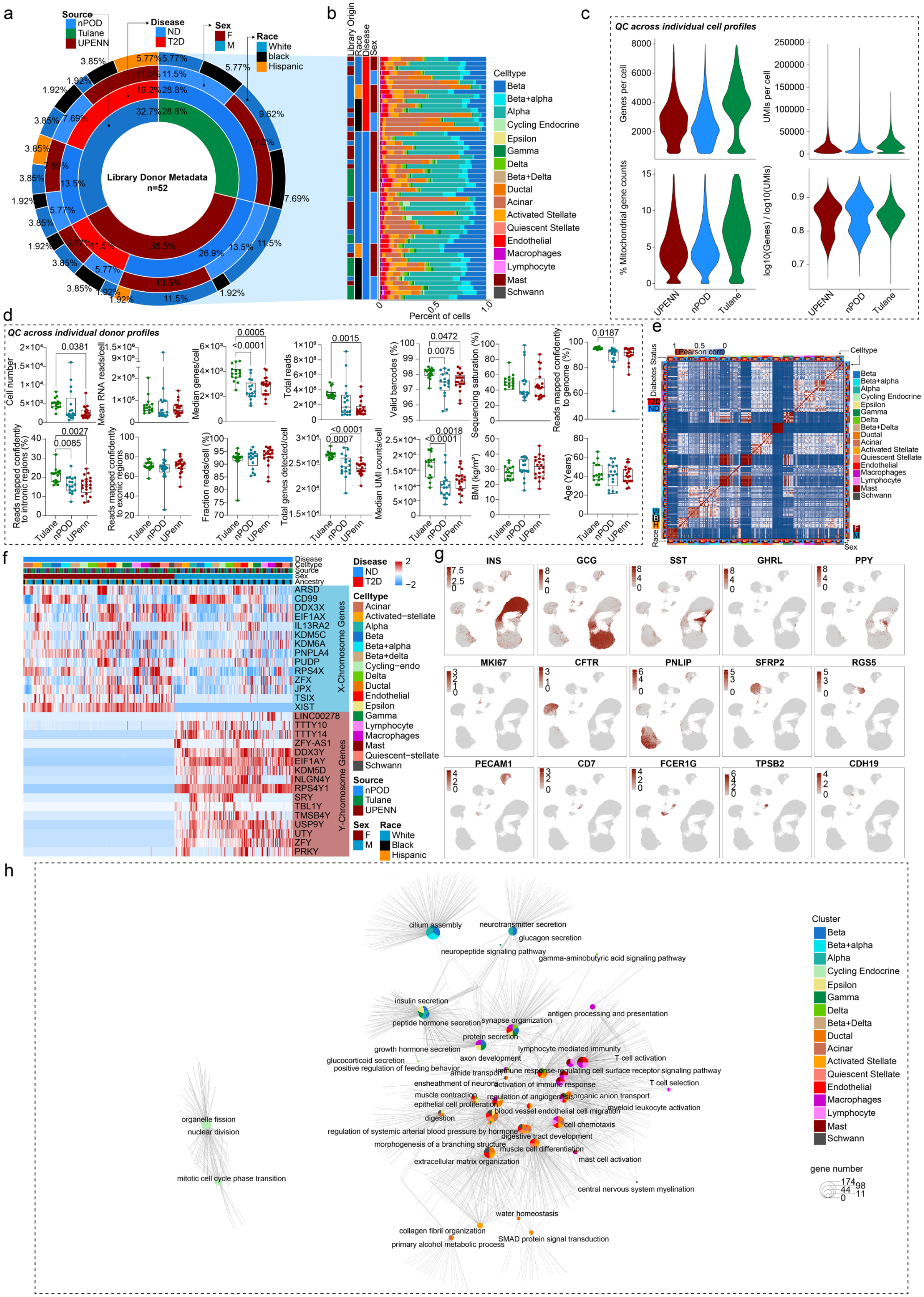
a
Source
nPOD
Tulane
UPENN
Disease
ND
T2D
Sex
F
M
Race
White
black
Hispanic
Library Donor Metadata
n=52
b
Library Origin
Race
Disease
Sex
Celltype
Beta
Beta+alpha
Alpha
Cycling Endocrine
Epsilon
Gamma
Delta
Beta+Delta
Ductal
Acinar
Activated Stellate
Quiescent Stellate
Endothelial
Macrophages
Lymphocyte
Mast
Schwann
Percent of cells
c
QC across individual cell profiles
Genes per cell
UMIs per cell
% Mitochondrial gene counts
log10(Genes) / log10(UMIs)
UPENN
nPOD
Tulane
d
QC across individual donor profiles
Cell number
Mean RNA reads/cell
Median genes/cell
Total reads
Valid barcodes (%)
Sequencing saturation (%)
Reads mapped confidently to genome (%)
Reads mapped confidently to intronic regions (%)
Reads mapped confidently to exonic regions (%)
Fraction reads/cell (%)
Total genes detected/cell
Median UMI counts/cell
BMI (kg/m²)
Age (Years)
Tulane
nPOD
UPenn
e
Pearson corr
Celltype
Diabetes Status
Race
Sex
f
Disease
Celltype
Source
Sex
Ancestry
X-Chromosome Genes
ARSD
CD99
DDX3X
EIF1AX
IL13RA2
KDM5C
KDM6A
PNPLA4
PUDP
RPS4X
ZFX
JPX
TSIX
XIST
Y-Chromosome Genes
LINC00278
TTTY10
TTTY14
ZFY-AS1
DDX3Y
EIF1AY
KDM5D
NLGN4Y
RPS4Y1
SRY
TBL1Y
TMSB4Y
USP9Y
UTY
ZFY
PRKY
Disease
ND
T2D
Celltype
Acinar
Activated−stellate
Alpha
Beta
Beta+alpha
Beta+delta
Cycling−endo
Delta
Ductal
Endothelial
Epsilon
Gamma
Lymphocyte
Macrophages
Mast
Quiescent−stellate
Schwann
Source
nPOD
Tulane
UPENN
Sex
F
M
Race
White
Black
Hispanic
g
INS
GCG
SST
GHRL
PPY
MKI67
CFTR
PNLIP
SFRP2
RGS5
PECAM1
CD7
FCER1G
TPSB2
CDH19
h
cilium assembly
neurotransmitter secretion
glucagon secretion
neuropeptide signaling pathway
gamma-aminobutyric acid signaling pathway
insulin secretion
peptide hormone secretion
synapse organization
antigen processing and presentation
protein secretion
growth hormone secretion
lymphocyte mediated immunity
glucocorticoid secretion
axon development
T cell activation
positive regulation of feeding behavior
amide transport
immune response-regulating cell surface receptor signaling pathway
ensheathment of neurons
activation of immune response
T cell selection
muscle contraction
regulation of angiogenesis
organic anion transport
epithelial cell proliferation
myeloid leukocyte activation
digestion
blood vessel endothelial cell migration
cell chemotaxis
regulation of systemic arterial blood pressure by hormone
digestive tract development
morphogenesis of a branching structure
muscle cell differentiation
extracellular matrix organization
mast cell activation
organelle fission
nuclear division
mitotic cell cycle phase transition
central nervous system myelination
water homeostasis
collagen fibril organization
SMAD protein signal transduction
primary alcohol metabolic process
Cluster
Beta
Beta+alpha
Alpha
Cycling Endocrine
Epsilon
Gamma
Delta
Beta+Delta
Ductal
Acinar
Activated Stellate
Quiescent Stellate
Endothelial
Macrophages
Lymphocyte
Mast
Schwann
gene number

**Appendix Figure S1. Donor metadata, scRNAseq quality control metrics and network analysis of all non-diabetic cell cells.**

**a,** Sunburst plot showing donor metadata of scRNAseq datasets used in this study. n=52 donors from Tulane University and the Human Pancreas Analysis Program (HPAP). Metadata is organized into source, disease status, sex, and race across all donor profiles. **b,** Percentage distribution across all 17 cell clusters for each of the n=52 donors used in this study. Cells are colored based on a key (legend) and metadata is organized based on information shown in **(a)**. **c,** Violin plots showing genes expressed per cell, unique molecular identifiers (UMIs) per cell, percentage mitochondrial genes and library complexity [log10(number of genes)/log10(number of UMIs)] across each of the donor sources. **d,** Quality control metrics for cell number, mean RNA reads/cell, median genes/cell, total reads, valid barcodes, sequencing saturation, percentage of reads mapped confidently to the reference genome, reads mapped confidently to intronic regions, reads mapped confidently to exonic regions, fraction of reads/cell, total genes detected/cell, median UMI counts/cell, basal metabolic index and age, across all donors split on the basis of source. **e,** Pearson's correlation between normalized read coverage within a merged set of RNAseq expression profiles across 52 donors. Annotation bars highlight the sex, race, cell type and diabetes status of the donors. **f,** Heatmap showing normalized aggregated RNAseq expression profiles for select Y and X linked chromosome genes across race, sex, source, celltype and disease. **g,** UMAP plots showing RNAseq expression profiles for select cognate genes across each cell type specific cell cluster. **h,** Gene ontology network analysis showing regulated pathways across all non-diabetic islet cells. Size of circle equivalent to gene contribution. **(d)** $p<0.05$ is significant **(h)** FDR adjusted $p<0.2$ is significant. Non-diabetic females: 20, non-diabetic males: 16, T2D females: 9, T2D males: 7.

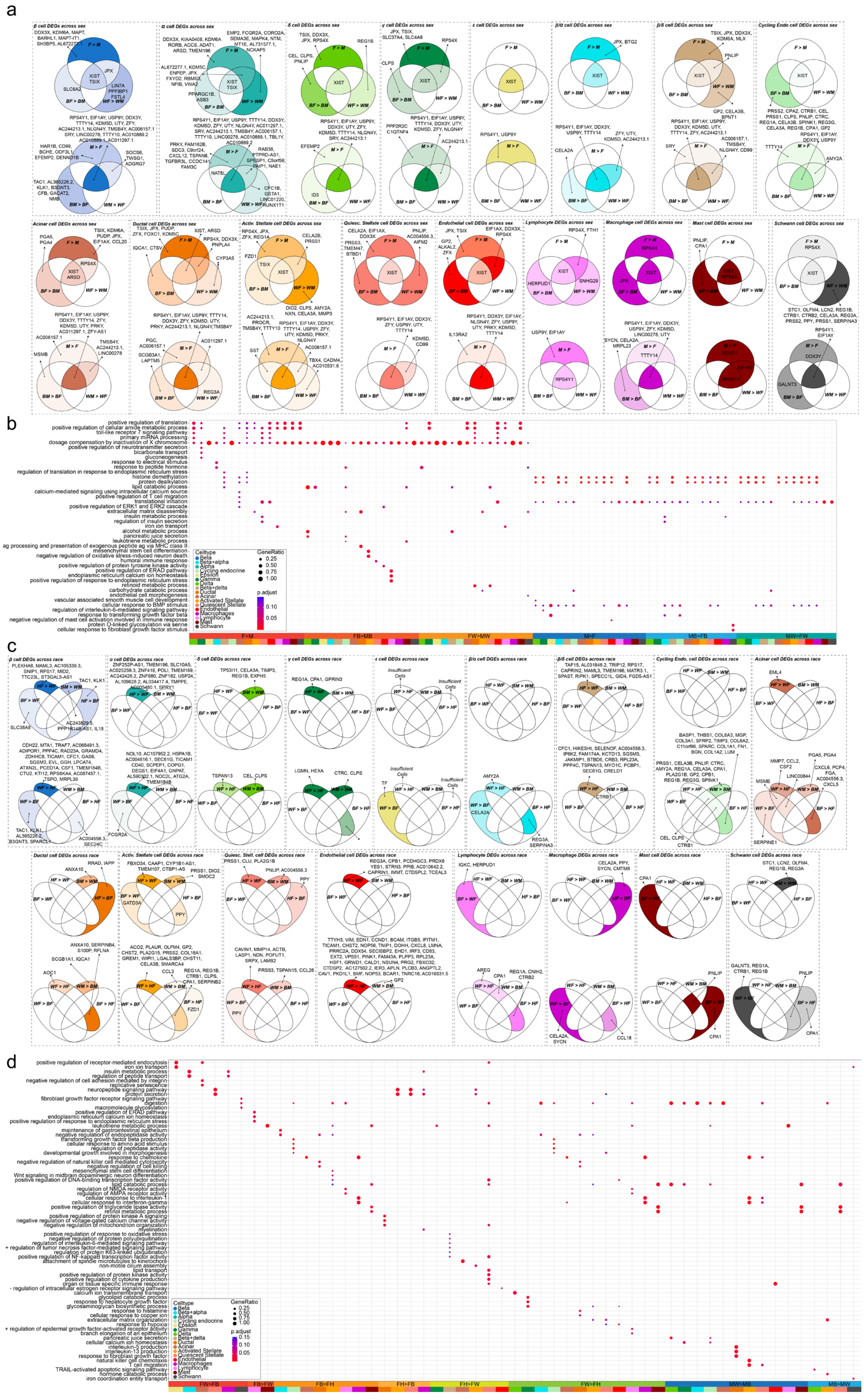
a
b
c
d

**Appendix Figure S2. Differentially expressed genes and pathways in human islet cells across sex and race.**

**a,** Venn diagrams showing differentially expressed genes (DEGs) across sex in case of non-diabetic: beta, alpha, delta, gamma, beta+alpha, beta+delta, cycling endo, acinar, ductal, activated stellate, quiescent stellate, endothelial, lymphocyte, macrophage, mast and schwann cells. **b,** Gene ontology (GO) analysis of celltypes other than α/β shown in **(a)**. **c,** Venn diagrams showing differentially expressed genes (DEGs) across ancestry for cells highlighted in **(a)**. **d,** Gene ontology (GO) analysis of all celltypes other than α/β shown in **(c)**. DGE: FDR adjusted pval < 0.1 is significant, GO: FDR adjusted pval < 0.2 is significant, Non-diabetic females: 20, non-diabetic males: 16.

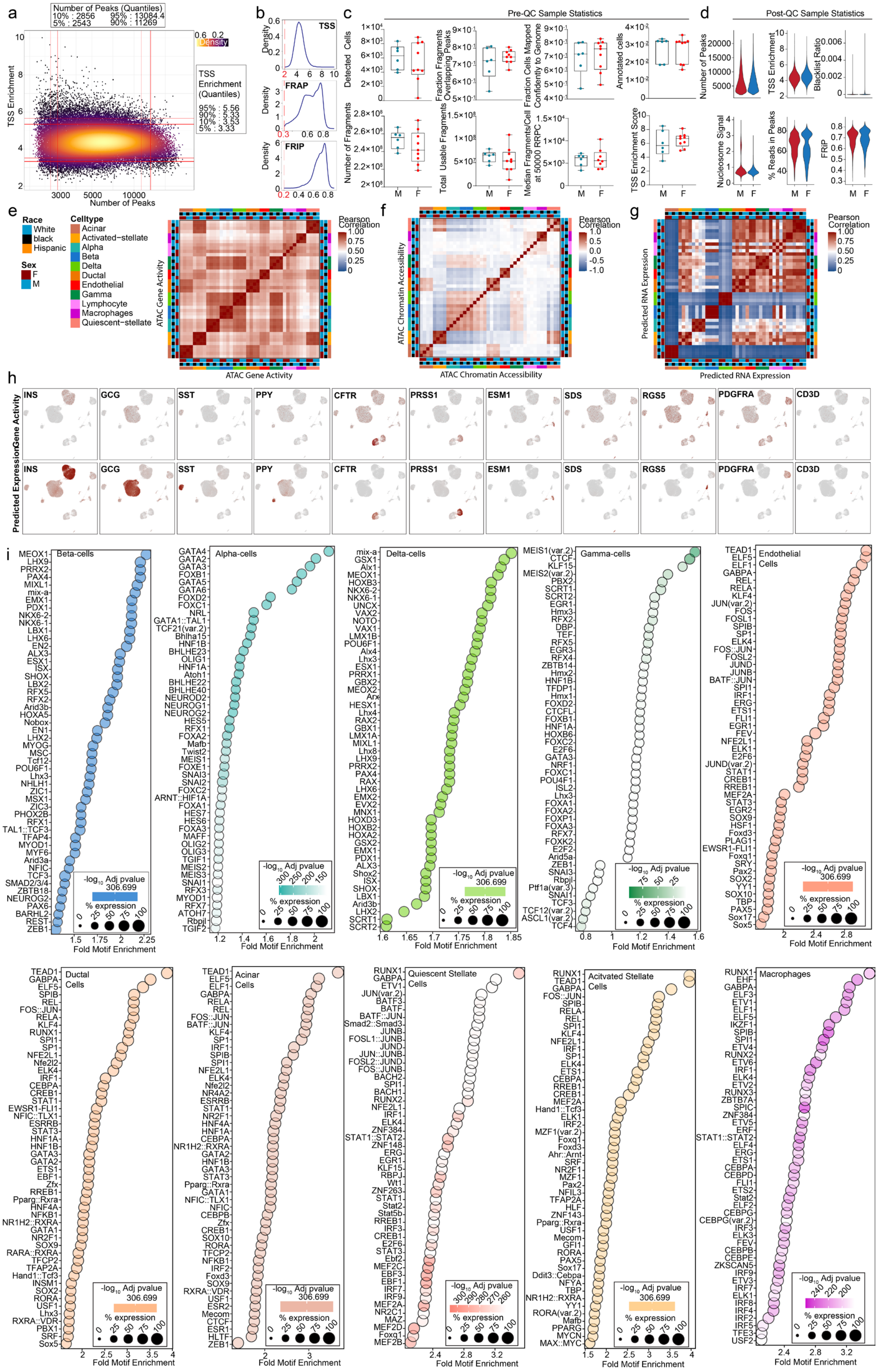
a
Number of Peaks (Quantiles)
10% : 2856 95% : 13084.4
5% : 2543 90% : 11269
TSS Enrichment (Quantiles)
95% : 5.56
90% : 5.33
10% : 3.53
5% : 3.33
Density
TSS Enrichment
Number of Peaks
b
TSS
FRAP
FRIP
Density
c
Pre-QC Sample Statistics
Detected Cells
Fraction Fragments Overlapping Peaks
Fraction Cells Mapped Confidently to Genome
Annotated cells
Number of Fragments
Total Usable Fragments
Median Fragments/Cell Fraction at 50000 RRPC
TSS Enrichment Score
M
F
d
Post-QC Sample Statistics
Number of Peaks
TSS Enrichment
Blacklist Ratio
Nucleosome Signal
% Reads in Peaks
FRIP
e
Race
White
black
Hispanic
Sex
F
M
Celltype
Acinar
Activated−stellate
Alpha
Beta
Delta
Ductal
Endothelial
Gamma
Lymphocyte
Macrophages
Quiescent−stellate
Pearson Correlation
ATAC Gene Activity
f
ATAC Chromatin Accessibility
g
Predicted RNA Expression
h
Gene Activity
Predicted Expression
INS
GCG
SST
PPY
CFTR
PRSS1
ESM1
SDS
RGS5
PDGFRA
CD3D
i
Beta-cells
Alpha-cells
Delta-cells
Gamma-cells
Endothelial Cells
Ductal Cells
Acinar Cells
Quiescent Stellate Cells
Activated Stellate Cells
Macrophages
-log10 Adj pvalue
% expression
Fold Motif Enrichment

**Appendix Figure S3. snATACseq quality control metrics and network analysis of all non-diabetic cells. a,** Density scatter showing transcriptional start site (TSS) enrichment on y-axis and number of peak counts on the x-axis. **b,** QC plots showing TSS, fragment reads in accessible peaks (FRAP) and fragment reads in promoters (FRIP) across all donors. Dashed red line signifies cutoff. **c,** QC plots showing metrics for detected cells, fraction fragments overlapping peaks, fraction cells mapped confidently to genome, annotated cells, number of fragments, total usable fragments, median fragments/cell at 50K raw reads/cell (RRPC) (cellranger did not calculate this metric for 1 female sample) and TSS enrichment score across all donors. **d,** Post-filtering QC metrics showing number of peaks, TSS enrichment, blacklist ratio, nucleosome signal, percentage reads in peaks and FRIP across all donors. **e,** Pearson's correlation between aggregated read coverage from snATACseq experiments. **f,** Pearson's' correlation between aggregated gene activity from snATACseq experiments. **g,** Pearson's' correlation between aggregated predicted RNA expression in snATACseq experiments based on mapped expression models from scRNAseq experiments. **h,** Gene activity based on accessibility in 100kb windows flanking select cognate genes across cell clusters (top row). Predicted gene expression based on expression data mapping from scRNAseq atlas (bottom row). **i,** Differential analysis using chromVAR and the JASPAR 2020 motif database showing enriched motifs for beta, delta, alpha, gamma, ductal, acinar, quiescent stellate, activated stellate, endothelial and macrophage cells. DGE: FDR adjusted pval < 0.05 is significantly enriched. Non-diabetic females: 9, non-diabetic males: 6.

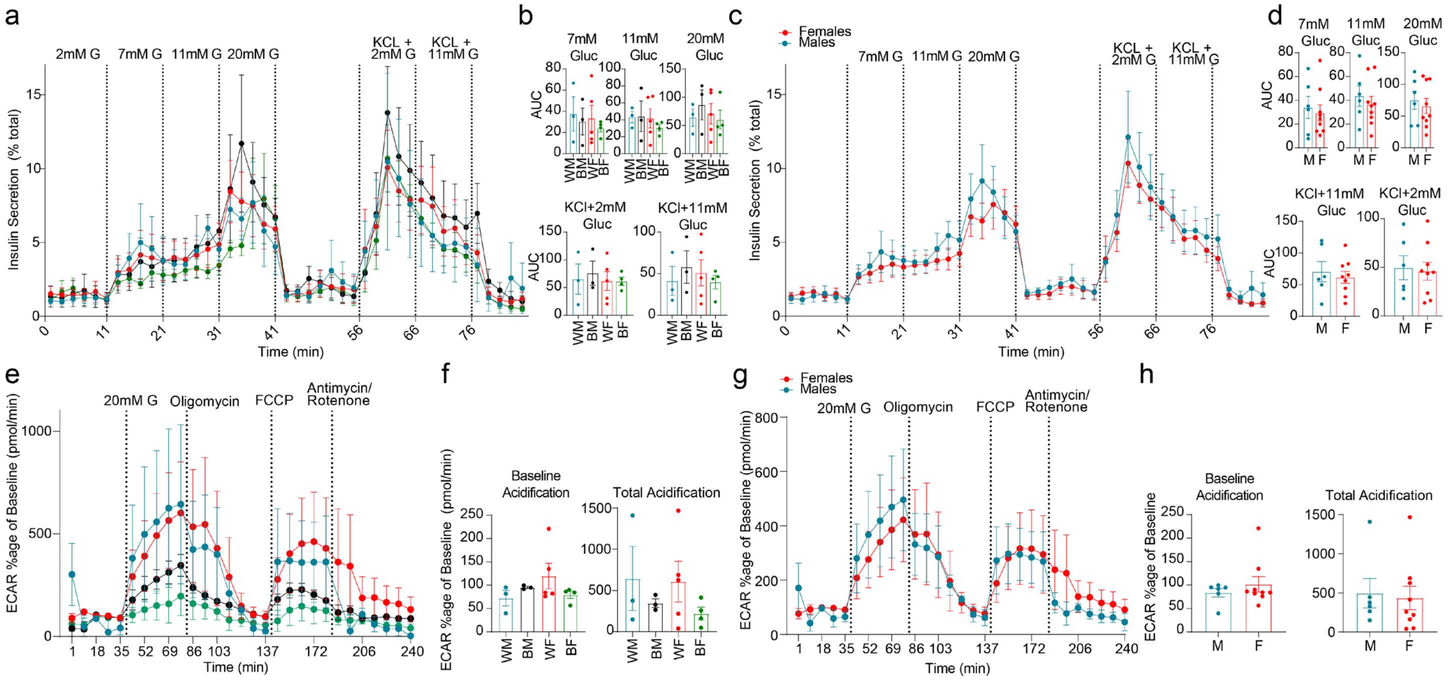


**Appendix Figure S4. Dynamic insulin and glucagon secretion coupled with islet bioenergetics.**

**a,** Dynamic insulin secretion assay using an ascending glucose ramp. Stimulation modes show response to 7mM glucose, 11mM Glucose, 20mM Glucose, potassium chloride + 2mM glucose and, potassium chloride + 11mM glucose. Each curve represents secretion normalized to total insulin content across sex and race. **b,** Area under the curve (AUC) measurements for incretin driven insulin secretion measurements outlined in **(a)**. **c,** Dynamic insulin secretion assay using an ascending glucose ramp. Stimulation modes show response to 7mM glucose, 11mM Glucose, 20mM Glucose, potassium chloride + 2mM glucose and, potassium chloride + 11mM glucose. Each curve represents secretion normalized to total insulin content across sex and race. **d,** Area under the curve (AUC) measurements for incretin driven insulin secretion measurements outlined in **(c)**. **e,** Extracellular acidification rate for islets across sex and race. **f,** Baseline acidification and total acidification of human islets across sex and race. **g,** Extracellular acidification rate for islets across sex. **h,** Baseline acidification and total acidification of human islets across sex. *pval < 0.05, **pval < 0.01 is significant. Non-diabetic females: 9, non-diabetic males: 6.

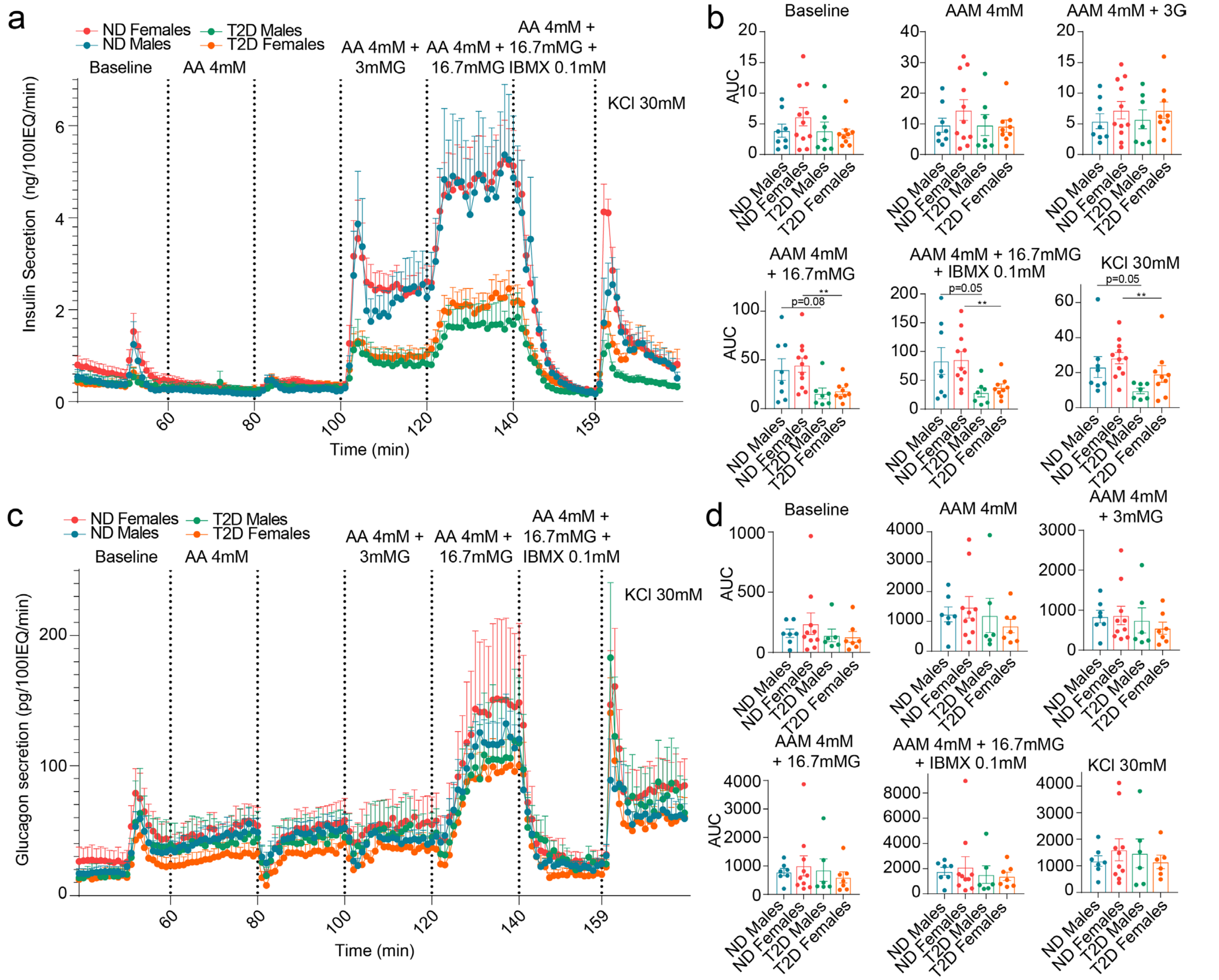


**Appendix Figure S5. Dynamic hormone secretion profiles of HPAP type 2 diabetic donors.**

**a,** Dynamic insulin secretion assay of human islets subject to Amino acid cocktail (AA) 4mM, AA 4mM + 3mM Glucose, AA 4mM +16.7mM glucose, AA 4mM + 16.7mM Glucose + IBMX 0.1mM and KCl 30mM. Each curve represents mean of each donor type across sex and disease. **b,** Area under the curve for insulin secretion at baseline, AA 4mM, AA 4mM + 3mM Glucose, AA 4mM +16.7mM glucose, AA 4mM + 16.7mM Glucose + IBMX 0.1mM and KCl 30mM. **c,** Dynamic glucagon secretion assay of human islets subject to secretagogues as in **(a)**. **d,** Area under the curve for glucagon secretion like **(a)**. Data analyzed from HPAP dataset. n=19 non-diabetic and n=16 T2D diabetic donors. For Glucagon measurements, n=17 non-diabetic and n=13 T2D diabetic donors. One donor lacked KCl data for both Insulin and Glucagon. Data is shown as mean±SE, bars show SE. **pvalue < 0.01.

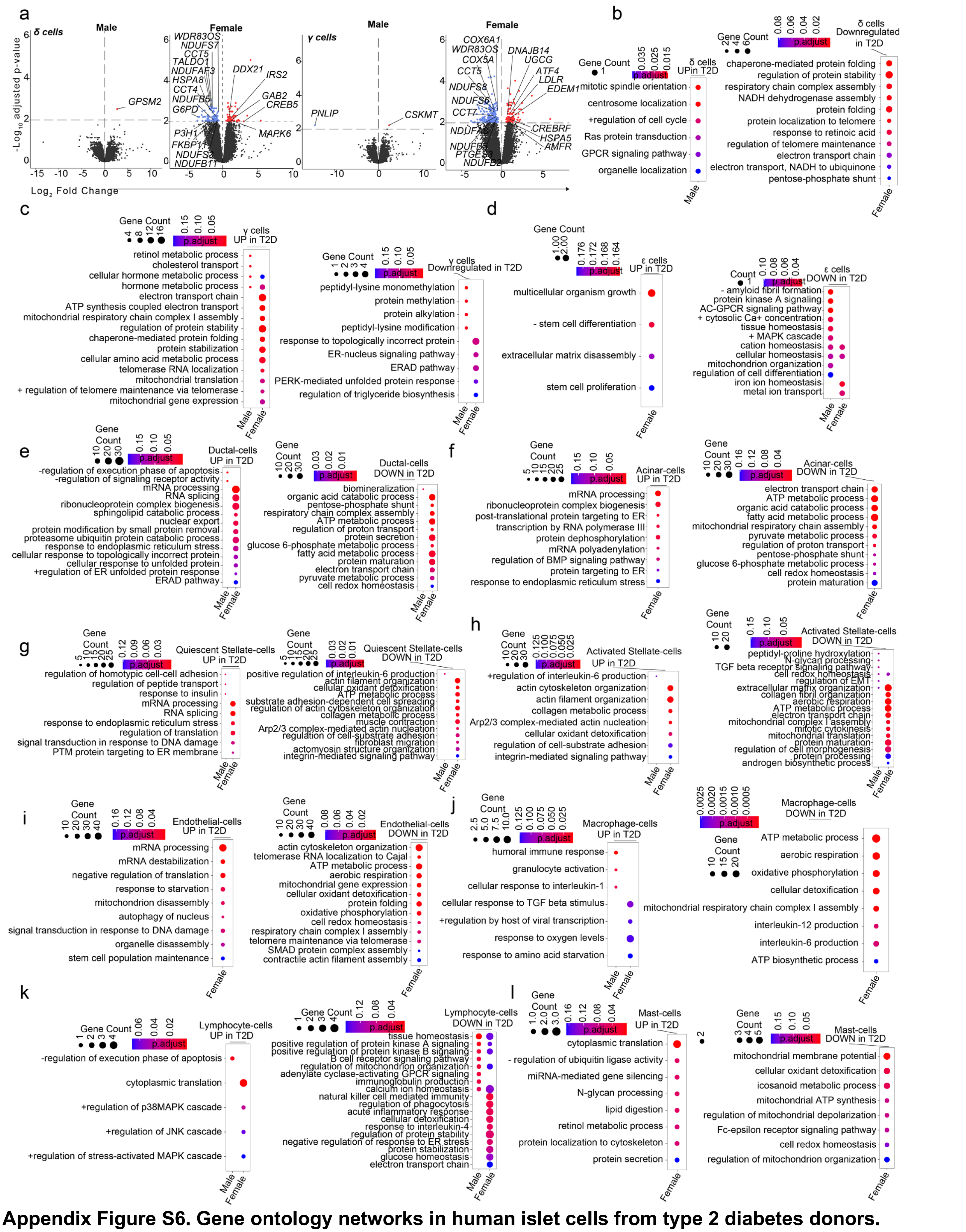

**Appendix Figure S6. Gene ontology networks in human islet cells from type 2 diabetes donors.**

**a-h,** Gene ontology plots highlighting upregulated and downregulated pathways in T2D across sex. Pathways compare type two diabetic males to non-diabetic males and similarly for females. Data is shown for: **a,** Ductal cells; **b,** Acinar cells; **c,** Quiescent stellate cells. **d,** Activated stellate cells; **e,** Endothelial cells; **f,** Macrophage cells; **g,** Lymphocyte cells; **h,** Mast cells. DGE: FDR adjusted pval < 0.1 is significant, GO: FDR adjusted pval < 0.2 is significant, Non-diabetic females: 20, non-diabetic males: 16, T2D females: 9, T2D males: 7.

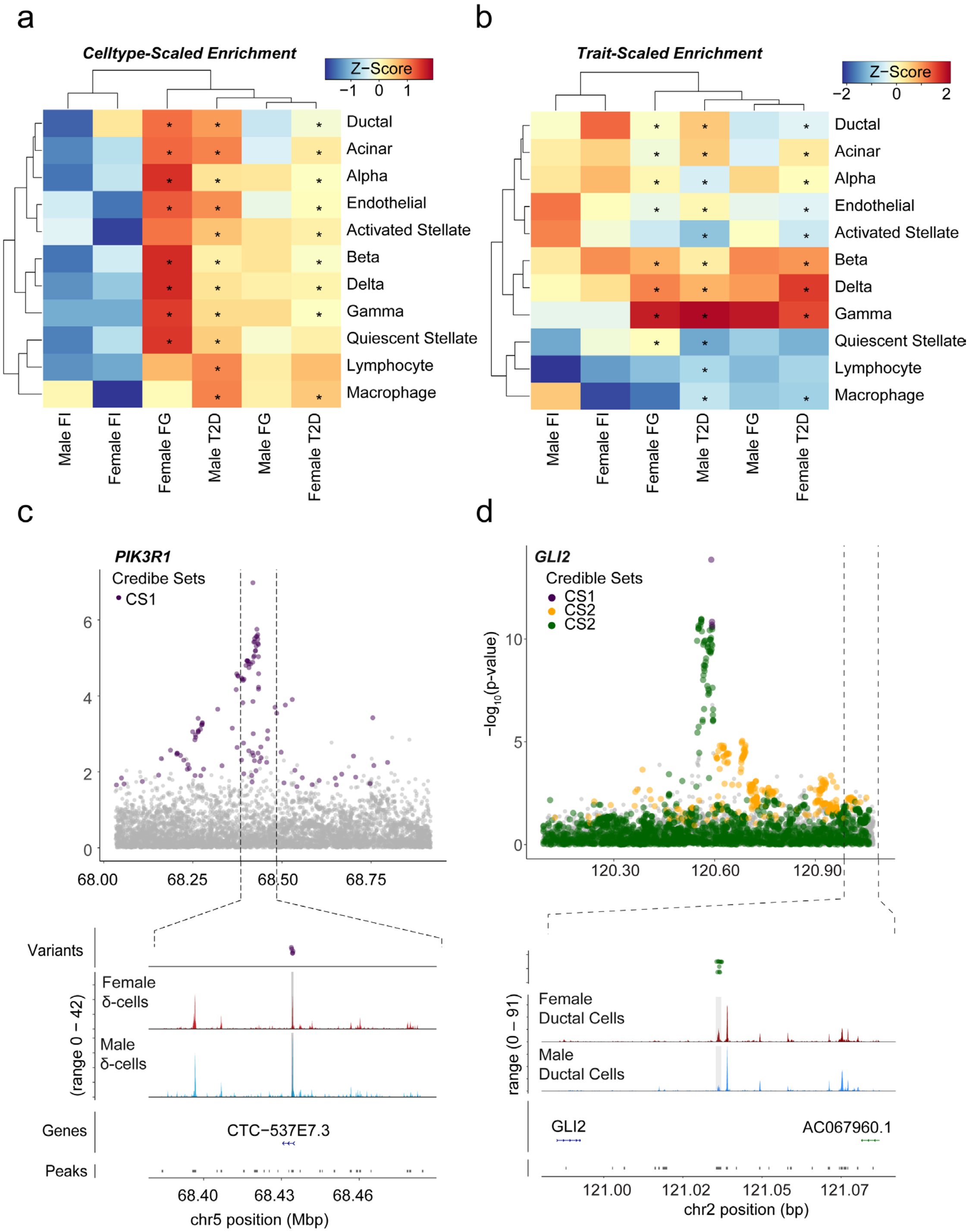


**Appendix Figure S7. Cell type-specific, GWAS using DIAMANTE T2D dataset. a-b,** Heatmaps showing cell type or trait scaled enrichment metrics for traits across sex and cell types identified in this study across the DIAMANTE T2D and MAGIC fasting insulin (FI) and fasting glucose (FG) GWAS datasets. **c,** Differentially accessible chromatin peak at chromosome PIK3R1: b38; 5:68433822–68434744 in male delta cells. **d,** Differentially accessible chromatin peak at b38; 2:121035441–121037137 in female ductal cells overlaps with credible set variants at T2D risk locus. Grey bars showing index variants overlapping with differentially accessible regions. male (N= 75,676) and female (N= 52,842) T2D GWAS.
